# Glycosaminoglycan analogs as a novel anti-inflammatory strategy

**DOI:** 10.3389/fimmu.2012.00293

**Published:** 2012-10-16

**Authors:** India C. Severin, Adriano Soares, Jennifer Hantson, Mauro Teixeira, Daniela Sachs, Delphine Valognes, Alexander Scheer, Matthias K. Schwarz, Timothy N. C. Wells, Amanda E. I. Proudfoot, Jeffrey Shaw

**Affiliations:** ^1^Merck Serono Geneva Research CentreGeneva, Switzerland; ^2^Departmento de Bioquimica e Imunologia, Instituto de Ciencias Biologicas, Universidade Federal de Minas GeraisBelo Horizonte, Brazil

**Keywords:** chemokine, chemokine antagonist, glycosaminoglycans, anti-inflammatory, NMR

## Abstract

Heparin, a glycosaminoglycan (GAG), has both anti-inflammatory and anti-coagulant properties. The clinical use of heparin against inflammation, however, has been limited by concerns about increased bleeding. While the anti-coagulant activity of heparin is well understood, its anti-inflammatory properties are less so. Heparin is known to bind to certain cytokines, including chemokines, small proteins which mediate inflammation through their control of leukocyte migration and activation. Molecules which can interrupt the chemokine-GAG interaction without inhibiting coagulation could therefore, represent a new class of anti-inflammatory agents. In the present study, two approaches were undertaken, both focusing on the heparin-chemokine relationship. In the first, a structure based strategy was used: after an initial screening of potential small molecule binders using protein NMR on a target chemokine, binding molecules were optimized through structure-based design. In the second approach, commercially available short oligosaccharides were polysulfated. *In vitro*, these molecules prevented chemokine-GAG binding and chemokine receptor activation without disrupting coagulation. However, *in vivo*, these compounds caused variable results in a murine peritoneal recruitment assay, with a general increase of cell recruitment. In more disease specific models, such as antigen-induced arthritis and delayed-type hypersensitivity, an overall decrease in inflammation was noted, suggesting that the primary anti-inflammatory effect may also involve factors beyond the chemokine system.

## Introduction

The process of inflammation involves the recruitment and activation of a wide variety of leukocytes. Chemokines are a large family of small proteins known to mediate this process. They activate target cells through a large family of seven transmembrane G-protein coupled receptors. *In vivo*, the situation is more complicated, and it has been proposed that chemokines work by producing immobilized or haptotactic gradients, which direct the migration of cells to the sites of inflammation, both by directing extravasation from the blood vessels and also migration through the tissues (Rot, 1993). These gradients are formed by the interaction of the chemokines with glycosaminoglycans (GAGs) (Handel et al., 2005). Studies on specific chemokines have mapped the binding sites of the GAGs to the chemokines, largely using heparin, and closely related GAGs such as chondroitin sulfate, and dermatin sulfate (Kuschert et al., 1998; Proudfoot et al., 2001; Lau et al., 2004). Modified chemokines, with both reduced and enhanced interaction between chemokines and GAGs, have been shown to modulate inflammatory processes (Johnson et al., 2004; Ali et al., 2005; Bedke et al., 2010; Tanino et al., 2010). Although protein therapeutics have been very successful for several indications, orally available small molecules could be more useful in long term therapies. Moreover, most therapeutic strategies address extracellular protein–protein interactions or intracellular signaling pathways. We have proposed interference with the chemokine/GAG interaction as a novel anti-inflammatory strategy, and have therefore tried two methods of identifying small molecules which can alter the chemokine-GAG interaction and reduce inflammation.

Heparin is a highly sulfated GAG which has a wide variety of molecular interactions. It displays promising anti-inflammatory activities clinically in asthma (Ahmed et al., 1993, 1999), ulcerative colitis (Evans et al., 1997), and burns (Iashvili et al., 1986). Heparin, as well as other GAGs, such as heparan sulfate (HS), also show potential as anti-cancer agents and a synthetic HS analog PI-88, which is an inhibitor of heparanase, is currently in clinical studies for treatment of lung cancer, liver cancer, as well as multiple myeloma and melanoma (Basche et al., 2006; Ferro et al., 2007; Kudchadkar et al., 2008).

However, its primary clinical action is to activate anti-thrombin III (AT III), via a conformational change, resulting in inhibition of both thrombin and factor Xa thereby preventing clotting. A specific pentapeptide sequence is required for this activity. Versions of low molecular weight heparin or analogs, without the anti-coagulant activities, have been produced to prevent erythrocyte resetting, a complication of malaria, (sevuparin, DF-02) and are in clinical phase I (Leitgeb et al., 2011).

Compounds that lack the anti-thrombotic effect of heparin, whilst maintaining its other activities, would be useful to further study the beneficial properties of GAGs in inflammatory and auto-immune diseases. We describe here two approaches to produce compounds that inhibit the chemokine activity, namely their binding to their receptors as well as to GAGs. Compounds that bind and neutralize chemokine ligands, as opposed to their receptors have been identified for the chemokine CXCL12 (Hachet-Haas et al., 2008; Galzi et al., 2010). We chose CCL5 as the chemokine target due to its well documented pro-inflammatory role in many diseases (Appay et al., 1999; Handel et al., 2005). The availability of a three dimensional structure, and the previous mapping of the GAG binding site (Chung et al., 1995; Shaw et al., 2004) was an important starting point for the design of specific molecules. The first approach was a structure based approach following identification of sulfated compounds that bind CCL5. The second was the sulfation of small oligosaccharides following the observation that 4-mer and 6-mer oligosaccharides inhibited CCL5-induced cellular recruitment into the peritoneal cavity (Shaw et al., 2004).

## Experimental procedures

### Reagents

Unless stated otherwise, all chemicals were purchased from Sigma Aldrich. The heparins used in the assays were heparin sodium salt from porcine mucosa (6–30 kDa; catalog number H3393) and low molecular weight heparin (3 kDa; catalog number H3400), both supplied by Sigma Aldrich. ^15^N-labeled CCL5 was prepared using standard procedures (Chung et al., 1995).

### Identification of CCL5 binders

An NMR-based screening approach was employed (Hajduk et al., 1999; Parish et al., 1999) to identify a small molecule binding to the chemokine CCL5. At concentrations required for NMR-based screening (>100 μM), CCL5 aggregates at physiological pH, and at pH values above 4.5 most of the proton resonances are severely broadened. Consequently, all experiments with wild-type CCL5 were performed at a pH 3.2 at 200 μM in 10% (v/v) deuterated water (D_2_O) and 90% (v/v) H_2_O, since in these conditions the protein is essentially a monomer.

A chemically diverse library composed of 206 compounds of low molecular weight (<350 Da) was designed for its potential ability to interact with GAG binding sites. These molecules were charged, and contained multiple acid groups, such as carboxylates and sulfonates. In addition, many of the molecules were aromatic, to provide some hydrophobicity. The library was pooled into sets containing five compounds and these pools mixed at 200 μM, with an equal concentration of CCL5 at 30°C, and a heteronuclear single quantum coherence (HSQC) spectrum recorded immediately. For pools that induced significant change in the protein chemical shifts, a second round of screening was performed in which each of the five compounds was added individually to the protein solution. For those molecules that displayed clear binding to CCL5, the dissociation constant *K*_*d*_ was measured by recording a series of ^15^N-HSQC spectra with increasing concentration of ligand. Dissociation constants were obtained by fitting the recorded chemical shift as a function of increasing ligand concentrations with:

(1) $$\delta_{\text{obs}}=\delta_{\text{f}}+L_{\text{b}}\Delta_{\text{b }-\text{f}}$$

in which δ_obs_ is the observed chemical shift at each point of the titration curve, δ_f_ is the chemical shift of the free protein and Δ_b − f_ is the difference in chemical shift between free and fully complexed protein.

### Structural determination of CCL5-binder complexes

**Figure 1:**
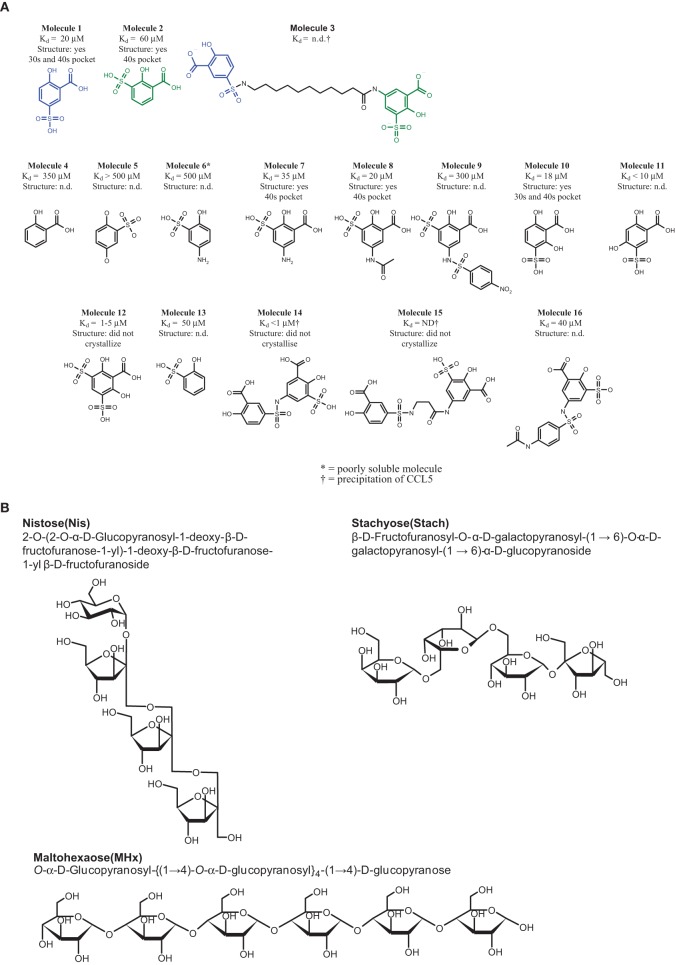
**Structure of the compounds. (A)** CCL5 binders. All the *K*_*d*_ values were determined by protein NMR. In the cases where the *K*_*d*_ could not be determined, either due to the low affinity of the compound, or due to precipitation of the complex, are indicated as not determined (n.d.). The molecules whose structure in complex with CCL5 was determined are indicated, including the pockets occupied by the molecule. Those molecules whose structure was attempted, but failed to crystallize are also described, while those for which no attempt to determined the co-crystal structure are indicated as not determined (n.d.). **(B)** The carbohydrates used for sulfation.

The crystallization conditions of CCL5 were essentially those previously described (Shaw et al., 2004). Briefly, CCL5 at 10 mg/ml in 50 mM acetate buffer pH 3.5, and the various molecules were incubated at a final concentration of the molecule of 0.1–0.5 mM (when solubility permitted) and crystallized at room temperature by hanging drop vapor diffusion in 15% (w/v) polyethylene glycol (PEG) 400, 50 mM acetate buffer pH 4.5, and 10% (w/v) glycerol. Crystallographic data were collected at 100 K on an Enraf-Nonius FR591 rotating anode generator equipped with Osmic MaxFlux mirrors and a MAR345 image plate detector. All the crystals of CCL5 belong to the orthorhombic space group P2_1_2_1_2_1_ with unit cell dimensions of *a* = 24 Å, *b* = 56 Å, and *c* = 94 Å, and contain a dimer of CCL5 in the asymmetric unit. Data was processed using DENZO and SCALEPACK (Otwinowski and Minor, 1997). Rigid body, simulating annealing, positional and B-factor refinement were performed with CNS (Brunger et al., 1998) and model building with Coot (Emsley et al., 2010). Bulk solvent and anisotropic B-factor corrections were applied. A number of other molecules, similar in structure to Molecule **1** were subsequently studied in the hope of establishing a structure-activity relationship (SAR), and in order to identify a promising starting point for the optimization of the CCL5-binders. In excess of 30 compounds, essentially poly-substituted phenyl sulfonates, were synthesized or purchased (see Figure 1A), and their binding affinity for CCL5 (*K*_*d*_ determined by NMR), and occasionally, their crystal structures determined (data not shown). It proved impossible to crystallize CCL5 in the presence of Molecule **3**, due to the propensity of this molecule to cause precipitation of CCL5, despite all attempts to maintain the complex in solution.

### Synthesis of the chimera

Molecule **3**: 5-[(11-{[(3-carboxy-4hydroxyphenyl) sulfonyl] amino} undecanoyl)amino]-2-hydroxy-3-sulfobenzoic acid.

The amide bond was formed with 1 equivalent of Molecule **2**, 1.5 equivalents of N-Boc aminoacid, 1.5 equivalents of N,N′-diisopropylcarbodiimide (DIC), and 2.5 equivalents of triethylamine (Et_3_N) in dichloromethane (DCM) overnight. A solution of 11-[(*tert*-butoxycarbonyl)amino]undecanoic acid (362 mg, 1.2 mmol) and DIC (151 mg, 1.2 mmol) in DCM was stirred at room temperature for 1 h. 5-amino-2-hydroxy-3-sulfobenzoic acid (233 mg, 1 mmol) and ET_3_N (202 mg, 2 mmol) were added, and the reaction mixture stirred overnight. Methanol and Amberlyst 15 were added and the reaction mixture stirred at room temperature for an additional 2 h. The solution was filtered and concentrated, produc-ing 5-({11-[(*tert*-butoxycarbonyl)amino]undecanoyl}amino)-2-hydroxy-3-sulfobenzoic acid at 84.8% purity by reversed-phase HPLC.

A solution of 5-({11-[(*tert*-butoxycarbonyl)amino] undecanoyl}amino)-2-hydroxy-3-sulfobenzoic acid in DCM/trifluoroacetic acid (9/1) was stirred at room temperature overnight. The solvents were removed to produce 5-[(11-aminoundecanoyl)amino]-2-hydroxy-3-sulfobenzoic acid as trifluoroacetate salt at 97.7% purity with an overall yield of 60% of the two steps (340 mg).

After removal of the Boc group, the sulfonamide bond was formed in dimethylformamide (DMF) with 10 equivalents of Et_3_N under vigorous stirring. A solution of 5-[(11-aminoundecanoyl)amino]-2-hydroxy-3-sulfobenzoic acid (250 mg, 0.3 mmol), 5-chlorosulfonyl-2-hydroxybenzoic acid (142 mg, 0.5 mmol) and triethylamine (304 mg, 3 mmol) in DMF was stirred at room temperature for 1 h. Methanol and Amberlyst 15 were added and the reaction mixture was stirred at room temperature for an additional 2 h. The solution was filtered and concentrated. The crude product was purified by preparative reversed-phase HPLC to produce the title compound at 50% yield (87 mg) and 100% purity.

### Synthesis of sulfated carbohydrates

The sulfation of commercially available sugars was performed as described earlier (Parish et al., 1999). Briefly, 100 mg of the starting material, namely Maltohexaose (MHx), Nistose (Nis) (1-O-(1-O-β-D-Fructofuranosyl-β-D-fructofuranosyl)-β-D-fructofuranosyl α-D-glucopyranoside, or Stachyose (Stach) (β-D-Fructofuranosyl-*O*-α-D-galactopyranosyl-(1→6)-*O*-α-D-galactopyranosyl-(1→6)-α-D-glucopyranoside (Figure 1B), were dissolved in 400 ml DMF and 600 ml pyridine, and 40 equivalents of sulfur trioxide-pyridine complex were added (642 mg for MHx or 955 mg for Nis and Stach). The reaction was stirred at 80°C for 3 h. The supernatant was decanted, the sticky yellowish residue was washed three times with methanol (MeOH), and then dissolved in 5 ml 0.1 M Tris/HCl buffer, pH 8.5. The resulting solution was extracted five times with 10 ml of ether (Et_2_O) to remove residual pyridine. The solution was then dialyzed twice against 5 L 20 mM Tris/HCl buffer pH 7.5, twice against 5 L 200 mM NaCl to make the sodium salt of the product and subsequently twice against 5 L double distilled water (ddH_2_O), using cellulose ester dialysis tubes with MWCO at 500 Da (Spectra/Por Biotech). The resulting persulfated oligosaccharides, namely persulfated MHx (MHxS), persulfated Nis (NisS), and persulfated Stach (StachS) were dried using a speed vacuum.

### Immobilized heparin competition binding assay

Competition experiments were performed as described elsewhere (Severin et al., 2010). Briefly, heparin-Sepharose beads (Sepharose beads or binding buffer as a background control) and increasing amounts of antagonist were added to each well of a 96 well filter plate. [^125^I]-CCL5, was added last, to reach a final concentration of 0.05 nM. The plates were incubated on a shaker at room temperature for 4 h. Each experiment was performed in triplicate, and the results are given as the mean of the three assays.

### Equilibrium competition receptor binding assays

The assays were carried out on membranes from CHO transfectants expressing CCR1 or CCR5, as described previously (Severin et al., 2010). Briefly, serial dilutions of the compounds were prepared in binding buffer (50 mM TrisHCl, pH 7.2, containing 1 mM CaCl_2_, 5 mM MgCl_2_, and 0.5% BSA). Equal volumes of membrane preparation at 80 μg/ml and scintillation proximity assay (SPA) beads were mixed before adding them to a 96 well filter plate, giving a final membrane concentration in the assay of 20 μg/ml. ^125^I labeled chemokines were dissolved in binding buffer at a concentration of 0.4 nM. The assay was started by the addition of 25 μL [^125^I]-CCL5 to give a concentration of 0.1 nM, in a final assay volume of 100 μL. The plates were incubated for 4 h. Radioactivity was counted with a beta counter for 1 min/well and the data analyzed using Graphpad Prism software. The radioligand was stored at −20°C and was used until the cpm of bound chemokine was ≥ 300 cpm.

### Anti-coagulation assay

Activated Partial Thromboplastin Time (APTT) assays were performed by GlycoMar. 10 mg/ml stock solutions of NisS, StachS, and MHxS, were prepared using dH_2_O and stored at −20°C until use. Samples were tested at 1 mg/ml. All further dilutions were in dH_2_O. Prior to each assay, a calibration curve of heparin ranging from 0 to 10 IU/ml was prepared using 5th International Standard Heparin. (GlycoMar ID:92, batch 97/578, Expiry 21SEP10). Fresh human blood from healthy human blood donors was collected into S-Monovette Coagulation tubes from Sarstedt. Nine parts of freshly drawn venous blood were collected into one part trisodium citrate (0.106 M). Plasma was obtained by centrifugation at 1500 × g for 10 min. Each of the carbohydrate samples was diluted and tested at 1 mg/ml in the first instance. MHxS was subsequently tested at 0.1 mg/ml to be within the time frame of the standard curve. The APTT assay was run on an ACL 9000 Coagulation Analyzer. The coagulation time of HemosIL Normal Control (cat no. 0020003110) was checked. Samples, standards and controls were mixed 1:10 with citrated plasma in a test tube, loaded into the ACL 9000 carousel and analyzed using the extended APTT run program.

### *In vivo* experimentation: animal welfare

All experimental protocols were carried out using international standards for animal experimentation and approved by the local authority where the experiment took place. More specifically, the mBSA-induced arthritis model was subjected to evaluation and approval by the animal ethics committee of the Universidade Federal de Minas Gerais (www.ufmg.br/bioetica/cetea/). As for the delayed-type hypersensitivity and the peritoneal recruitment models, all applied procedures were approved by and respected the best practices for animal studies promoted by the Animal Experimentation Domain of the General Health Direction of the Republic and Canton of Geneva (Republique et Canton de Genève, Direction Générale de la Santé, Domaine de l'expérimentation animale). In general, animals were acclimatized for at least 1 week prior to experimentation, maintained at 12/12 h light/dark cycle and given food and water ad libitum. The mouse strain that was used and its origin is referred to in the description of each model below.

All compounds were tested for endotoxin content prior to administration.

### Peritoneal recruitment

CCL5-induced peritoneal recruitment was performed as described (Johnson et al., 2004). Thioglycollate-induced cellular recruitment was mediated by the administration of 40 mL/kg of 3% thioglycollate i.p. in 7- to 8-week-old female C3H/Hen mice (JANVIER). Sham mice were injected with 40 mL/kg of NaCl (0.9%, LPS free). Saline (vehicle, control group) or Maltohexaose Sulfate at doses ranging from 1 to 0.01 mg/kg diluted in a volume ratio of 10 mL/kg and were administered i.p. 30 min before the thioglycollate stimulus, and 24 h later. An additional group of mice was injected with a single dose of 3 mg/kg dexamethasone 1 h before challenge as a reference control. Mice were sacrificed 48 h post-thioglycollate injection, the peritoneal cavity was washed twice with 5 mL of 0.5 M EDTA solution in PDB, pH 7.4. The peritoneal lavage fluid was centrifuged and resuspended in 1 mL of the solution and recruited cells were counted with a cell counter (Beckman-Coulter, ACT5Diff AD32097).

### Delayed-type hypersensitivity (DTH) model

Female BALB/c mice were immunized with 2 × 10^6^ sheep red blood cells (SRBC) by subcutaneous route (*s.c*.) at the base of the tail. Five days later mice were challenged with an *s.c*. injection of 2 × 10^6^ SRBC into the softpaw of the left hindpaw. The paw thickness was measured 21 h later and the difference calculated based on values obtained before challenge. MHxS, NisS, and StachS were injected *s.c*. at a dose of 10 mg/kg, 30 min before and 8 h after challenge. Vehicle was 0.02% BSA diluted in PBS. The reference compound, dexamethasone, was diluted in saline and administered by oral gavage 30 min before challenge. Group sizes were 6 for sham and 8 for all other groups.

### Antigen-induced arthritis (AIA) model

Induction of disease was performed by injection of 10 μg methylated bovine serum albumin (mBSA) as a 10 μL injection into the synovial cavity. Test compounds, at a total dose of 50 μg were injected in a total volume of 200 μL subcutaneously immediately after mBSA administration. Group sizes were 4–6 animals. Total leukocytes (A), neutrophils (B), and mononuclear cells (C) in the synovial cavity were enumerated 24 h after mBSA injection (Sachs et al., 2011).

### Statistical analysis

The results from the peritoneal recruitment models-induced by CCL5, thioglycollate, the DTH model, and the knee cavity recruitment were analyzed by One-Way-ANOVA followed by Newmann-Keuls multiple comparisons test.

## Results

### Identification of CCL5 binders

15 compounds of the 206 compounds screened elicited a change in the protein chemical shifts, but only three of these, Molecules 1, 8, and 10 were confirmed to be selectively binding to CCL5; the most striking being Molecule **1** (See Figure 1A). The chemical shift perturbations observed for the amide resonances of Thr43, Arg44, and Lys45 in the presence of Molecule **1** suggest that the compound binds in the 40s loop of CCL5.

### Analysis of the binding mode of CCL5 binders identified by NMR and X-ray crystallography

**Figure 2:**
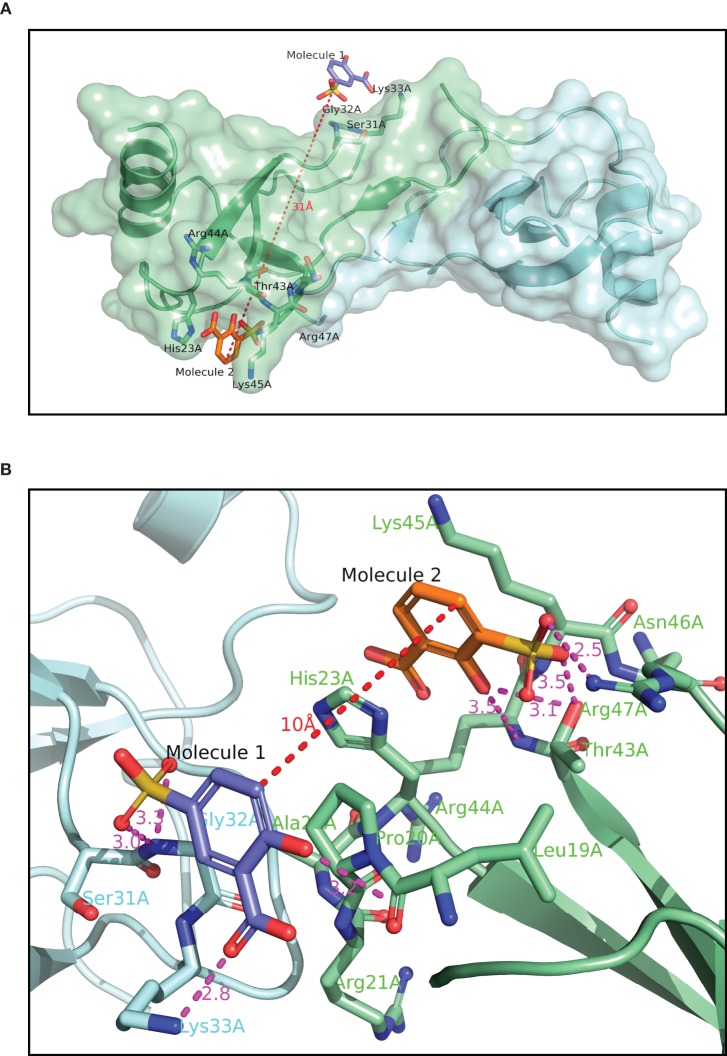
**Crystallographic structure of CCL5 (A) with Molecules 1 and 2 bound.** The contents of the asymmetric unit are displayed, showing the distance between the two molecules binding to the protein. **(B)** binding site of Molecules **1** and **2**. The binding pocket for Molecule **2** is the same as in panel 2A, but Molecule **1**, and its associated binding pocket are from a symmetry-related dimer. The distance between the two molecules in thus only 10 Å, compared to over 30 Å in panel 2A.

The structure of Molecule **1** complexed to CCL5 was obtained at a resolution of 1.8 Å (see Figure 2A). The complex crystallized in the same crystal form as the wild-type protein, with two monomers (called monomer A and B) in the asymmetric unit. With the exception of the extreme N- and C-termini, the protein structure is essentially the same as that of the wild-type protein. However, the analysis of the structure revealed that the two copies of Molecule **1** in the structure were not identical, and that one of the two molecules was actually a minor contaminant (<0.5%) of the original batch of Molecule **1**. Molecule **1** was found in close proximity to a surface loop composed of residues Ser31A-Gly32A-Lys33 (hereafter called the 30s pocket; see Figure 2A). The sulfonate group of Molecule **1** forms two hydrogen bonds with the main chain nitrogen of Gly32A (3.0 Å and 3.3 Å), while the carboxylate group of Molecule **1** forms a hydrogen bond with the Lys33A sidechain (2.8 Å). The hydroxyl group of Molecule **1** forms a hydrogen bond with a crystallographic symmetry-related monomer of CCL5 to the main chain carbonyl group of Pro18A (3.2 Å). Molecule **2** (see Figure 1A) is the minor contaminant identified in the crystal structure, and binds to a pocket on monomer A composed of residues Thr43A to Arg47A (hereafter called the 40s pocket; see Figure 2B). The sulfonate group of Molecule **2** forms a hydrogen bond with Thr43A (2.5 Å), and a relatively weak one with Arg47A side chain (3.5 Å). The hydroxyl group of Molecule **2** forms a hydrogen bond with Thr43A (3.1 Å). While in the same asymmetric unit, Molecules **1** and **2** are fairly distant from one another (~25 Å), they are relatively close when a crystallographic symmetry-related molecule of CCL5 is included (~10 Å). It is this proximity that suggested that the linking of Molecules **1** and **2** might engender a molecule with much higher potency than either of the individual molecules, since they may be acting as two-independent GAG monomers. Consequently, attempts were made to optimize Molecules **1** and **2** for their respective pockets, and subsequently to link them.

Numerous Molecule **1** or **2** analogs were synthesized and tested for their affinity to CCL5, and the crystal structures of several of them were determined, in order to identify their binding site (see Figure 1A). Many of the synthesized molecules bound to either the 30s loop, or the 40s pocket, as expected. Some of the new molecules bound to both sites (data not shown). The analysis of the SAR of the analogs of Molecule **1** suggested that the presence and position of the sulfonate group is absolutely required for binding to the 30s pocket, but that this sulfonate group may be substituted to produce various sulfonyl groups. The study of the SAR of Molecule **2** revealed that the substitution pattern of Molecule **2** cannot be varied, but that substitution at the para position of the hydroxyl group is tolerated. The determination of the relative affinities of these Molecules **1** and **2** analogs was complicated by the fact that the more potent compounds-induced precipitation of CCL5, rendering the affinity measurement by NMR impossible.

In a further attempt to increase the potency of the individual binders identified by NMR screening, the two Molecules **1** and **2** were chemically linked together in order to produce a chimera, Molecule **3**. After several attempts, comprising liquid- and solid-phase strategies the amide bond formation was achieved using the Boc-protected amino acid in the presence of DIC using Et_3_N as the base. The overall synthesis was hampered by the relatively low solubility of the final product, giving an overall yield of 30%.

### Inhibition of binding by the CCL5 binders

**Figure 3:**
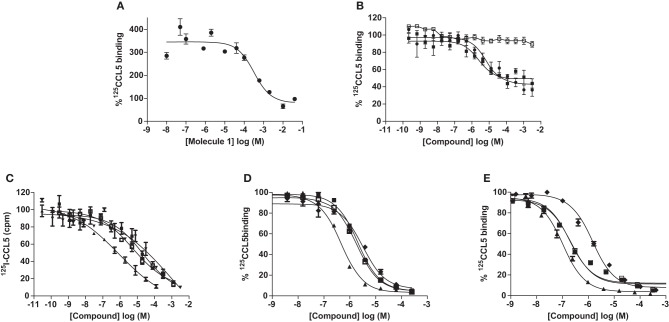
**Inhibition of CCL5 binding by GAG analogs.** Inhibition of CCL5 binding to heparin was measured by the ability to compete for ^125^I-CCL5 binding to heparin beads by Molecule **1 (A)** and persulfated oligosaccharides **(C)**. Inhibition of CCL5 binding to its receptors was determined by competition equilibrium binding assays using membranes from transfectants expressing CCR1 **(B)** and **(D)** or CCR5 **(E)** by persulfated oligosaccharides. Legend: • Molecule **1**; ■ Molecule **3**; □ Molecule **1** on CCR5; ♦ MHxS; ▼ StachS; ▲ NisS; ○, 3 kDa (H3400) heparin.

A study of the relative affinity of Molecule **1** toward CCL5 and heparin molecules was first evaluated by competition experiments in which CCL5, immobilized on heparin beads, was competed off the beads by increasing concentrations of Molecule **1**. In this experiment, an IC_50_ of 0.32 mM for Molecule **1** was observed (Figure 3A). Despite this low IC_50_, Molecule **1** is the first small molecule identified capable of disrupting the interaction between heparin and CCL5. Since the principal GAG binding motif of CCL5, the BBXB motif on the 40s loop, plays a role in binding to CCR1, we determined the capacity of Molecule **1** to inhibit the binding of the chemokine CCL5 to its receptors CCR1 and CCR5 using equilibrium competition binding with a SPA-based assay. Molecule **1** was in fact more potent in this assay, as it competed ^125^CCL5 for binding to CHO/CCR1 transfectants with an IC_50_ value of 6.7 μM, but had no effect on CCL5 binding to CCR5, consistent with the fact that the GAG binding motifs of CCL5 are distinct from those for CCR5 (Figure 3B). In order to evaluate if Molecule **1** could be a general CCR1 antagonist, the same experiment was performed with CCL3 as the ligand; however, no competition of CCL3 to its receptors CCR1 and CCR5 was observed (data not shown). We conclude that Molecule **1** is selective for CCL5 binding to CCR1. However, contrary to our predictions, Molecule **3** demonstrated no increased potency as it demonstrated an IC_50_ value of 2.5 μM for inhibition of binding to CCR1 (Table 1, Figure 3B).

**Table 1:** **IC_50_ values (μM) of GAG analogs for the inhibition of CCL5 binding to heparin and CCL5 receptors**.

	**Heparin**	**CCR1**	**CCR5**
**CCL5 BINDERS**			
Molecule **1**	320	6.7	n.a.
Molecule **2**	nd	nd	nd
Molecule **3**	nd	2.5	nd
**PERSULFATED OLIGOSACCHARIDES**			
NisS	5.89 ± 0.04	1.92 ± 1.31	0.18 ± 0.02
StachS	18.6 ± 4.34	1.47 ± 0.89	0.20 ± 0.01
MHxS	1.27 ± 0.67	0.49 ± 0.31	0.03 ± 0.03
3 kDa heparin	22.6 ± 3.25	1.58 ± 0.63	0.48 ± 0.67

nd, not determined; n.a., no activity.

### Inhibition of binding by the sulfated GAG analogs

As the sulfated oligosaccharides are structurally closely related to heparin fragments, we tested their ability to inhibit the chemokine heparin interaction. Using the CCL5 heparin bead binding assay, we detected similar IC_50_ values for all three sulfated oligosaccharides with MHxS being the most potent (Table 1 and Figure 3C) and all were superior compared to the value obtained for fractionated 3 kDa (H3400) heparin.

We again used the SPA binding assay to test the inhibition of receptor binding by the sulfated GAG analogs, in comparison to 3 kDa heparin (H3400). All three of the sulfated compounds were potent inhibitors of CCL5 binding to both CCR1 and CCR5, in contrast to the synthetic molecules such as Molecule **1** (Table 1 and Figures 3D,E).

### Anti-coagulation assay

We performed APTT assays to determine the anticoagulant properties of the tetrasaccharides NisS and StachS and the hexasaccharide MHxS in comparison to heparin and heparin-derived tetrasaccharide and hexasaccharide pools (dp4 and dp6, respectively). No anti-coagulation activity was found for the dp4 and dp6, whilst the anticoagulant potency was 12-fold reduced for MHxS and 15-fold reduced for NisS and StachS compared to heparin (Table 2).

**Table 2:** **Anti-coagulation activity**.

**Compound**	**Coagulation time (IU/mg)**
Heparin	152.9 ± 26.2
dp4	0.97 ± 0.30
dp6	2.03 ± 0.65
NisS	6.87 ± 3.22
StachS	6.90 ± 2.69
MHxS	11.87 ± 3.07

### Thioglycollate-induced peritoneal recruitment assays

**Figure 4:**
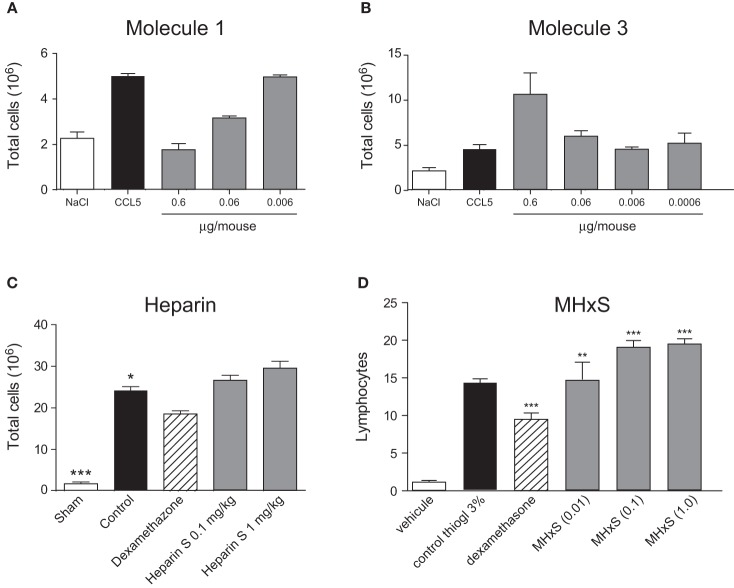
**Inhibition of peritoneal cellular recruitment.** Cellular recruitment into the peritoneal cavity was induced by CCL5 **(A)** and **(B)** and inhibited by different doses of CCL5 binders administered 30 min prior to the CCL5 administration. Cellular recruitment into the peritoneal cavity was induced by thioglycollate **(C)** and **(D)** and treated by increasing doses of heparin **(C)** and MHxS **(D)** using dexamethasone as a positive control. The statistical differences versus the control groups (CCL5 alone-treated mice in **A** and **B**; or Thioglycollate alone-treated mice in **C** and **D**) are shown whenever present and indicated by ^*^P < 0.05; ^**^P < 0.01 or ^***^P < 0.001.

In order to determine, if the inhibition of heparin and receptor binding produces an anti-inflammatory effect, we used a simple *in vivo* model of inflammation. The model we chose was thioglycollate-induced peritonitis. As shown in Figure 4, despite its lack of potency in the *in vitro* assays, Molecule **1** inhibited cell recruitment to the peritoneum in a dose dependent manner. However, when we tested Molecule **3** designed to bind to two separate sites of CCL5, we observed an enhancement of cell recruitment to the peritoneal cavity (Figure 4B).

The sulfated GAGs also displayed increased recruitment in this peritonitis assay. Again, rather than inhibit cell recruitment the compounds enhanced recruitment, albeit moderately, as demonstrated for MHxS (Figure 4D). In contrast to previously published results for CCL5 mediated peritonitis (Shaw et al., 2004), heparin showed a small tendency to enhance recruitment in this model (Figure 4C). We hypothesized that endogenous cells in the peritoneal cavity might be released upon injection of heparin and the compounds, thus enhancing the number of cells in the lavage, which was incorrectly interpreted as enhanced recruitment. We therefore performed peritoneal washes with solutions containing either heparin or the compounds. We found that neither heparin nor the GAG-related compounds increased the number of cells in these washes compared to a peritoneal wash with PBS or PBS/EDTA (data not shown).

### Delayed-type hypersensitivity

**Figure 5:**
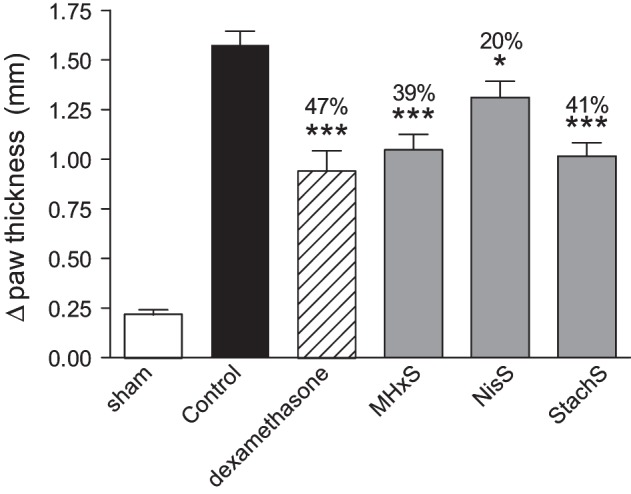
**Inhibition of delayed-type hypersensitivity.** Immunization was performed by *s.c.* administration of SRBC, followed by a challenge in the hindpaw 5 days later. The persulfated oligosaccharides (10 mg/kg) were administered 30 min prior and 8 h after the challenge. Dexamethasone was used as a positive control, and paw thickness was measured 21 h following the challenge. The statistical differences vs. the control group (mice challenged with SRBC and treated with vehicle for the compounds 0.02% BSA diluted in PBS) are shown whenever present and indicated by ^*^P <0.05>; ^***^P < 0.001.

Despite the fact that the peritoneal recruitment assay led to enhanced recruitment, we tested the anti-inflammatory potential of the sulfated GAG analogs in another model, delayed-type hypersensitivity. Initial experiments were performed in order to define the optimal dose and the regimen for the administration of MHxS. A dose-response experiment using 0.1, 1, and 10 mg/kg (dosing at −30 min and at +8 h post-challenge) was performed and statistically significant inhibitions were shown only for the highest dose (data not shown). In a subsequent experiment we tested whether we could alter the efficacy of MHxS in this model by varying the frequency of administrations while keeping the same dose of 10 mg/kg. We concluded that while the pre-administration of MHxS at −30 min was important for the observed effect, the administration of a pre-treatment dose 24 h before challenge on top of the dosings performed at −30 min and at +8 h did not further improve the inhibition in paw thickness-induced by MHxS. Finally, a head to head comparison of the efficacy of NisS, StachS, and MHxS was performed at the best conditions defined in earlier experiments, i.e., fixed dose of 10 mg/kg with compounds being administered at −30 min and +8 h post-challenge. These results are shown in Figure 5, where paw swelling was inhibited by treatment with NisS, StachS, and MHxS by an order of 20, 41, and 39% respectively in comparison to 47% for dexamethasone.

### Antigen-induced arthritis model

**Figure 6:**
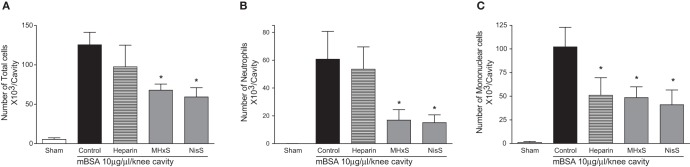
**Inhibition of antigen induced arthritis.** An injection of mBSA into the synovial cavity was used to induce disease. 50 μg of the inhibitors were administered *s.c.* immediately after the mBSA injection, and the cells in the synovial cavity were enumerated 24 h later. **(A)** Total cells; **(B)** lymphocytes; **(C)** mononuclear cells. ^*^P < 0.05.

The sulfated GAG compounds also displayed anti-inflammatory properties in mBSA-immunized mice, a rodent model for arthritis. We found a statistically significant reduction of neutrophil and mononuclear cell recruitment into the knee cavity after treatment with MHxS or NisS, and a reduction of mononuclear cells after treatment with heparin (Figure 6).

## Discussion

We have described two approaches for the generation of molecules with potential anti-inflammatory activity as a substitute for heparin. In the first approach, we linked two small molecules that were identified by NMR screening and X-ray crystallography to bind to two separate sites on the chemokine CCL5 to form a chimera with potentially more potent inhibitory activity than the separate molecules. In the second approach, we persulfated commercially available short carbohydrates in order to obtain sulfated GAG analogs with the anti-inflammatory properties of heparin but lacking the anti-coagulant activity.

Molecule **1** (Figure 1A) presents the first small molecule described to inhibit the interaction of CCL5 with heparin with an IC_50_ of 320 μM, and is also able to prevent the interaction of CCL5 with the receptor CCR1. This molecule was capable of preventing the recruitment of leukocytes to the peritoneum of the mouse, with an EC_50_ of 0.06 μg/ mouse. Molecule **2** was identified from the NMR screen, and so would be expected to have a similar affinity for CCL5. Direct measurement of the affinity was not possible because the protein complex precipitated. Molecule **2** was found to bind to CCL5 from the X-ray crystallographic structure, but at a different site, closer to the dimer interface. Based on the identification of these two different binding sites, Molecule **3** was synthesized, which contains the key active functional groups of Molecules **1** and **2**, with an appropriate spacer. However, we were not able to demonstrate a more potent inhibition of these interactions with Molecule **3**. We were not able to measure an interaction constant for Molecule **3** with CCL5, due to the precipitation issues. There was an encouraging signal in the CCR1 binding assay, where Molecule **3** showed a 3-fold tighter interaction than Molecule **1**. *In vivo*, however, there was no inhibition of recruitment, rather an enhancement at the highest dose. Our working hypothesis is that Molecule **3** is able to crosslink CCL5 molecules, thereby forming higher order oligomers of CCL5, which are known to be responsible for the activity in the murine model (Proudfoot et al., 2003). Since CCL5 exists under physiological conditions as an oligomer (Bruhl et al., 2001) the precise molecular interaction of Molecule **3** would either have to stabilize the active conformation, or provide additional affinity sites, in order to have a biological effect. The difficulty of predicting the *in vivo* activity of compounds from the *in vitro* data and structural analysis suggested that this line of investigation was unlikely to generate a therapeutically useful molecule.

In the search for anti-inflammatory compounds which would block the chemokine GAG interaction, we performed the study on persulfated GAG analogs. These analogs were chosen based on the length, since anything longer than six saccharides proved impossible to study in the structural and biochemical assays, and anything shorter than four saccharides lacked sufficient potency for meaningful biological studies. All three persulfated compounds showed weak micromolar activity in the inhibition of heparin binding, but showed sub-micromolar activity in blocking CCR1 binding, and an order of magnitude more potent in blocking CCR5. However, MHxS also enhanced recruitment in the peritoneal recruitment assay, as was observed for Molecule **3**.

The role of GAG binding was first demonstrated to be essential for the activity of chemokine-induced cell migration *in vivo* (Proudfoot et al., 2003). Recent studies have, however, shown that abrogation of GAG binding can result in an increased cellular recruitment in certain tissues (Tanino et al., 2010). Although mice lack CXCL8, the human chemokine is capable of inducing neutrophil recruitment in mice; mutants of CXCL8 which have lost their capacity to bind to heparin, nonetheless recruit more neutrophils than the wild type protein when instiled into the lungs. It was also observed that recruitment-induced by the murine neutrophil CXC chemoattractants KC and MIP-2 gave different results, with KC being the most efficient, despite the fact that surface plasmon resonance had demonstrated that KC associated and dissociated more rapidly from surface bound heparin. These data suggest that the relationship between chemokines and cell surface GAGs is in fact more complex than initially suspected, and will require a more thorough investigation of this biology, particularly in view of targeting this axis as a possible anti-inflammatory strategy. Interestingly it has recently been shown that GAGs can also show opposing effects *in vitro*, where CXCL8 mediated chemotaxis was inhibited by heparin but induction of reactive oxygen species was enhanced by several GAG families (Schlorke et al., 2012).

Despite the conflicting results in the simple *in vivo* model of peritoneal cellular recruitment, we subsequently tested the GAG analogs in two other *in vivo* models of inflammation. The first model was a delayed-type hypersensitivity model in mice immunized with sheep red blood cells, and the second was an antigen-induced arthritis model where mice were immunized with mBSA. In both of these models, we could demonstrate a beneficial effect of the sulfated oligosaccharides. In the DTH model MHxS and StachS showed a comparable effect on the reduction of paw thickness as dexamethasone which was used as a control treatment (Figure 6). In the mBSA-induced arthritis model both MHxS and NisS reduced the recruitment of neutrophils and mononuclear cells to the knee cavity, whereas heparin only had an effect on the recruitment of mononuclear cells (Figure 6).

It remains to be elucidated why these compounds have an anti-inflammatory effect in these two models, but an apparent pro-inflammatory effect in the simple peritoneal recruitment assay. There are some differences in these models that could contribute to these contradictory effects. The peritoneal recruitment assay is performed in naïve mice, whereas for the DTH and AIA models the mice are previously immunized. This implies that in these models the immune response is elicited by the adaptive immune system, whereas in peritoneal recruitment it is a response of the innate system. It should also be noted that the CXCL12 binder showed inhibition of the inflammatory infiltrate in an allergic airway inflammation model of, again a model elicited by the adaptive immune system (Hachet-Haas et al., 2008).

To support the idea of searching for novel compounds that interfere with the chemokine-GAG axis, it should not be forgotten that heparin has been used as an anti-coagulant in the clinic for decades and a pro-inflammatory effect has never been documented. Since heparin also causes increased recruitment into the peritoneal cavity, we could conclude that this is a peculiarity of this compartment.

In conclusion, we have demonstrated that the identification of compounds interfering with chemokine-GAG binding is possible. These compounds display anti-inflammatory activity, but further work will be required to unravel the mechanism of action that provides both the pro-inflammatory and anti-inflammatory effects *in vivo*. The inhibitory effect of the compounds under inflamed conditions could be attributed to several mechanisms, but predominantly to disruption of the chemokine-GAG interaction. Further work should be undertaken to determine the affinity of the compounds to GAGs expressed in the physiological context, such as heparan sulphate, dermatan sulphate, or chondroitin sulphate. However, other mechanisms could play a role such as inhibition of CCR1 mediated recruitment or possible prevention of the formation of oligomeric states of the chemokine which have been shown to be essential for cellular recruitment mediated by certain chemokines *in vivo* (Proudfoot et al., 2003). Lastly, since the oligosaccharide family provides the highest diversity among biological macromolecules (Shriver et al., 2002), providing a wealth of possible structures, these compounds could potentially provide a novel class of therapeutics and the concept of GAG mimetics has in fact been explored for the inhibition of chemokine-induced metastasis (Sutton et al., 2007; Friand et al., 2009).

### Conflict of interest statement

The authors declare that the research was conducted in the absence of any commercial or financial relationships that could be construed as a potential conflict of interest.
